# Application of Petri net based analysis techniques to signal transduction pathways

**DOI:** 10.1186/1471-2105-7-482

**Published:** 2006-11-02

**Authors:** Andrea Sackmann, Monika Heiner, Ina Koch

**Affiliations:** 1 Technical University of Applied Sciences Berlin, Bioinformatics group, Seestr. 64, 13347 Berlin, Germany; 2 Poznań University of Technology, Institute of Computing Science, ul. Piotrowo 2, 60-965 Poznań, Poland; 3 Brandenburg University of Technology at Cottbus, Dept. of Computer Science, Postbox 10 13 44, 03013 Cottbus, Germany; 4 Friedrich-Schiller-University Jena, Institute of Computer Science, Ernst-Abbe-Platz 2, 07743 Jena, Germany

## Abstract

**Background:**

Signal transduction pathways are usually modelled using classical quantitative methods, which are based on ordinary differential equations (ODEs). However, some difficulties are inherent in this approach. On the one hand, the kinetic parameters involved are often unknown and have to be estimated. With increasing size and complexity of signal transduction pathways, the estimation of missing kinetic data is not possible. On the other hand, ODEs based models do not support any explicit insights into possible (signal-) flows within the network. Moreover, a huge amount of qualitative data is available due to high-throughput techniques. In order to get information on the systems behaviour, qualitative analysis techniques have been developed. Applications of the known qualitative analysis methods concern mainly metabolic networks. Petri net theory provides a variety of established analysis techniques, which are also applicable to signal transduction models. In this context special properties have to be considered and new dedicated techniques have to be designed.

**Methods:**

We apply Petri net theory to model and analyse signal transduction pathways first qualitatively before continuing with quantitative analyses. This paper demonstrates how to build systematically a discrete model, which reflects provably the qualitative biological behaviour without any knowledge of kinetic parameters. The mating pheromone response pathway in *Saccharomyces cerevisiae *serves as case study.

**Results:**

We propose an approach for model validation of signal transduction pathways based on the network structure only. For this purpose, we introduce the new notion of *feasible *t-invariants, which represent minimal self-contained subnets being active under a given input situation. Each of these subnets stands for a signal flow in the system. We define *maximal common transition sets *(*MCT-sets*), which can be used for t-invariant examination and net decomposition into smallest biologically meaningful functional units.

**Conclusion:**

The paper demonstrates how Petri net analysis techniques can promote a deeper understanding of signal transduction pathways. The new concepts of feasible t-invariants and MCT-sets have been proven to be useful for model validation and the interpretation of the biological system behaviour. Whereas MCT-sets provide a decomposition of the net into disjunctive subnets, feasible t-invariants describe subnets, which generally overlap. This work contributes to qualitative modelling and to the analysis of large biological networks by their fully automatic decomposition into biologically meaningful modules.

## Background

Signal transduction pathways are of special interest in biological and medical sciences. Many diseases are related to disturbances in signalling pathways. For example protein-tyrosine kinases (PTKs) are important regulators of intracellular signal transduction pathways, mediating development and multicellular communication in metazoans. Their activity is tightly controlled and regulated, but perturbation of the normal autoinhibitory constraints on kinase activity can result in oncogenic PTK signalling [1]. Another example are human G-protein-coupled receptors, which mediate response to light, odour, taste, hormones, and neurotransmitters. Widely prescribed drugs, such as *β*-adrenergic receptor blockers, antihistamines, and serotonin-reuptake inhibitors bind to specific G-protein-coupled receptors [2]. There are signalling modules, e.g., this G-protein and the mitogen-activated protein-kinase (MAPK) cascade, which occur in eukaryotic organisms from yeast to human. Budding yeast (*Saccharomyces cerevisiae*) has proven to be indispensable in understanding the mechanisms, interrelationships, and regulation of these components. On account of this crucial role of budding yeast we discuss our approach on the best understood signalling pathway in *S. cerevisiae*, the one of the mating pheromone response [3,4].

Signal transduction pathways are usually modelled by a set of ordinary differential equations to describe the dynamic changes in the concentrations of the involved biochemical species [5-8]. One of the main problems in using these quantitative methods is the incomplete knowledge of kinetic parameters. Often only 30–50% of them are known, such that existing methods for parameter estimation fail. In addition, the numerical values of concentrations of species may not be considered, if they are very small, because of rounding effects in the solver algorithms. No insight into possible signal flows and the connections between species, e.g., feedback loops, are given explicitly. Moreover, more qualitative than quantitative data became available in the last years. This all has initiated the development of qualitative methods, i.e., discrete models and related evaluation techniques, which are used as a complementary intermediate step for construction and understanding of larger reliable models.

In order to model and analyse biochemical pathways on a qualitative level, dedicated methods have been developed. An example for a well established concept are the elementary modes [9], which are based on the incidence (stoichiometric) matrix of the underlying directed graph. This approach is applied for analysing metabolic networks. They are considered to be in steady state in order to use convex cone analysis techniques.

Recently, interaction graphs and Boolean networks are used to model and analyse signalling and regulatory networks [10]. [11] shows the limits of logical modelling techniques for gene regulatory networks. The authors propose an integrative systematic approach of logical and Petri net modelling discussing the tryptophan biosynthesis in *E. coli*. However, analyses are mentioned only in the outlook. Opposite to [12], who hold the view that the concept of elementary modes or minimal t-invariants, respectively, cannot be applied to signalling networks, [10] uses this concept, as we had already proposed in [13-15] to analyse an apoptosis model.

We have based our modelling approach of signalling pathways on Petri net theory, because it comprises several analysis techniques including those, which are based on the incidence matrix. In contrast to the above mentioned techniques, Petri nets provide useful unique visualisation techniques with possibilities for hierarchical modelling and for animation. Especially this aspect is of crucial importance for experimentalists and for the dialog between experimentalists and theoreticians.

Carl Adam Petri introduced the basic concepts of Petri nets in his dissertation in the context of technical systems [16]. He developed an approach for describing and studying models, which consist of concurrent, i.e., independent, and/or causally dependent components. Since this time, many theorems and algorithms have been developed and implemented to analyse Petri net models, see, e.g., [17,18]. First applications of Petri net theory to biological systems were published in [19-21]. Meanwhile, metabolic pathways [22,23], signal transduction pathways [14,24], and gene-regulatory networks [25,26] have been modelled and analysed successfully using various classes of Petri nets, qualitative as well as quantitative ones. An overview is given, e.g., in [27,28]. For an earlier bibliography of related application papers see [29].

Signal transduction pathways exhibit some special properties. In contrast to metabolic networks, in signalling pathways there is no substance flow, but a signal flow, which is performed, for example, by phosphorylated and dephosphorylated protein forms. There are no stoichiometric reaction equations given, which dictate the net structure and define the arc weights. Modelling signalling pathways we have to work on another abstraction level than in modelling metabolic networks. Petri nets provide a generic description principle, applicable on any level of abstraction.

In this paper we present a method, which enables the systematic deduction of the net structure from the known signalling processes in the cell, which are translated into logical terms as implication, conjunction, inclusive or exclusive disjunction, and negation. These terms can be translated unambiguously to Petri net components. The resulting model is a qualitative, discrete Petri net, which can be analysed for certain system properties to validate the model.

In the following, we give a brief introduction into the mating pheromone response pathway in *S. cerevisiae *and recall the necessary Petri net definitions. Then, we propose and discuss in detail a systematic modelling method with respect to signalling pathways. We focus on the analysis of signal flows and introduce for this purpose the new concepts of *feasible *t-invariants and of *maximal common transition sets *(*MCT-sets*). Feasible t-invariants stand for minimal self-contained subnets being active under a given input situation, while MCT-sets can be interpreted as smallest biologically meaningful functional units. Both concepts define a fully automatic net decomposition into biologically meaningful modules. Whereas MCT-sets provide a decomposition of the net into disjunctive subnets, feasible t-invariants describe subnets, which generally overlap. We apply our modelling technique to the analysis of the pheromone response pathway in *S. cerevisiae *and discuss the results. Finally, we give conclusions indicating also further projects.

### The signal transduction pathway of mating pheromone response

The signalling pathway of the mating pheromone response in *S. cerevisiae *is reviewed, e.g., in [3,4,30]. In *S. cerevisiae *there are two different mating types of haploid cells, MAT*α *and MAT**a**. Cells of opposite mating type can mate, i.e., fuse to a diploid cell. This process is stimulated through small peptide pheromones, the *α*-factor and the **a**-factor, respectively. A haploid cell secretes the own factor and carries receptors for detecting a cell of opposite mating type via the other factor. When a yeast cell is stimulated by pheromone, secreted by a nearby cell of the opposite mating type, it starts a complex signalling pathway to undergo a series of physiological changes in preparation for mating. These changes are controlled and regulated through the pheromone signalling pathway, which is nearly the same in MAT*α *and MAT**a **cells.

In the following, we refer to this pathway in MAT**a **cells, so the considered cells have an *α*-factor-specific cell surface receptor STE2, which is coupled to a heterotrimeric G-protein. This G-protein consists of the three subunits G*α*, G*β*, and G*γ*, whereby the latter two can act as heterodimer G*βγ*. A conformation change of the receptor catalyses an exchange of GDP to GTP in the G*α *subunit leading to a dissociation of this monomer. If GTP is hydrolysed to GDP, the monomer reassociates with the dimer to the trimeric form of the G-protein [30]. The G-protein G*βγ *subunits mediate a signal on the MAP kinase cascade by interacting with Cdc24 (Far1 transmitted), protein kinase Ste20, and scaffold protein Ste5. The Ste20, Ste11, and Ste7 kinases, which are activated serially, phosphorylate and activate itself the two MAP kinases Fus3 and Kss1. But, Fus3 attenuates the activation of Kss1 [31]. Fus3PP releases the transcription factor Ste12 of its suppression through Dig1 and Dig2 proteins. (Re-)Forming such a repression complex necessitates inactive Fus3 or inactive Kss1 [32]. Pheromone induced genes prepare the mating of the two cells and the fusion of their nuclei. They are responsible for cell cycle arrest in the phase G1, and the synthesis of some signalling regulators, e.g., the protease Bar1, the regulator of G-Protein Sst2, and the phosphatase Msg5. Other regulations of the signalling pathway are realised through receptor endocytosis [33] or degradation of involved kinases [34].

### Petri nets

Let us first recall some basic definitions of Petri nets. More formal definitions are given, e.g., in [17,18,35]. Petri nets are bipartite directed multi-graphs, i.e., they consist of two types of nodes, called places *P *= {*p*_1_,..., *p*_*n*_} and transitions T = {*t*_1_,..., *t*_*m*_}, and directed arcs, which are weighted by natural numbers and connect only nodes of different type. Places model typically passive system elements as conditions, states, or biological species, i.e., chemical compounds as proteins. Transitions stand generally for active system elements as events, or chemical reactions as de-/activation. In graphical representations, places are depicted as circles and transitions as rectangles. Transitions without preplaces (postplaces) are called input (output) transitions and are drawn as flat rectangles. The arcs in the net describe the causal relation between active and passive elements. They are illustrated as arrows and labelled with their weight, if it is larger than one. Arcs connect an event with its preconditions, which must be fulfilled to trigger this event, and with its postconditions, which will be fulfilled, when the event takes place.

The fulfilment of a condition is realised via tokens residing in places. Principally, a place in a discrete net may carry any integer number of tokens, indicating different degrees of fulfilment. If all preplaces of a transition are marked sufficiently (corresponding to the arc weights) with tokens, this transition may fire. If a transition fires, tokens are removed from all its preplaces and added to all its postplaces, each corresponding to the given arc weights. Thus, the tokens are the dynamic elements of the system. If a condition must be fulfilled, but the firing of an adjacent transition does not remove any tokens from it, these nodes are connected via two converse arcs. In the following, these arcs are represented by bidirectional arrows as a short-hand notation and are called *read arcs*.

From a biological viewpoint, the tokens residing in places indicate whether the corresponding chemical species is present, i.e., its concentration is above a certain concentration level (threshold) in the cell. This presence enables the chemical reactions modelled by the place's posttransitions to take place.

A current distribution of the tokens over all places, usually given as **m **∈ ℕ0n
 MathType@MTEF@5@5@+=feaafiart1ev1aaatCvAUfKttLearuWrP9MDH5MBPbIqV92AaeXatLxBI9gBaebbnrfifHhDYfgasaacH8akY=wiFfYdH8Gipec8Eeeu0xXdbba9frFj0=OqFfea0dXdd9vqai=hGuQ8kuc9pgc9s8qqaq=dirpe0xb9q8qiLsFr0=vr0=vr0dc8meaabaqaciaacaGaaeqabaqabeGadaaakeaatuuDJXwAK1uy0HMmaeHbfv3ySLgzG0uy0HgiuD3BaGabaiab=vrionaaDaaaleaacqaIWaamaeaacqWGUbGBaaaaaa@39D7@, describes a certain system state and is called a *marking *of the net. Accordingly, the *initial marking ***m**_0 _of a net describes the system state before any transition has fired.

The incidence matrix *C *of a given Petri net is an (*n *× *m*)-matrix (where *n *denotes the number of places and *m *the number of transitions, compare above). Every matrix entry *c*_*ij *_gives the token change on the place *p*_*i *_by the firing of the transition *t*_*j*_. Thus, the incidence matrix does not reflect read arcs. A t-invariant is defined as a non-zero vector *x *∈ ℕ0m
 MathType@MTEF@5@5@+=feaafiart1ev1aaatCvAUfKttLearuWrP9MDH5MBPbIqV92AaeXatLxBI9gBaebbnrfifHhDYfgasaacH8akY=wiFfYdH8Gipec8Eeeu0xXdbba9frFj0=OqFfea0dXdd9vqai=hGuQ8kuc9pgc9s8qqaq=dirpe0xb9q8qiLsFr0=vr0=vr0dc8meaabaqaciaacaGaaeqabaqabeGadaaakeaatuuDJXwAK1uy0HMmaeHbfv3ySLgzG0uy0HgiuD3BaGabaiab=vrionaaDaaaleaacqaIWaamaeaacqWGTbqBaaaaaa@39D5@, which holds the equation

*C*·*x *= 0.     (1)

A t-invariant represents a multiset of transitions, which have altogether a zero effect on the marking, i.e., if all of them have fired the required number of times, a given marking is reproduced. The invariant property holds for an arbitrary initial marking. A t-invariant is called *realisable*, if a marking is reachable, such that all transitions of the t-invariant are able to fire in a suitable partial order. Analogously, a p-invariant is defined as a non-zero vector *y *∈ ℕ0n
 MathType@MTEF@5@5@+=feaafiart1ev1aaatCvAUfKttLearuWrP9MDH5MBPbIqV92AaeXatLxBI9gBaebbnrfifHhDYfgasaacH8akY=wiFfYdH8Gipec8Eeeu0xXdbba9frFj0=OqFfea0dXdd9vqai=hGuQ8kuc9pgc9s8qqaq=dirpe0xb9q8qiLsFr0=vr0=vr0dc8meaabaqaciaacaGaaeqabaqabeGadaaakeaatuuDJXwAK1uy0HMmaeHbfv3ySLgzG0uy0HgiuD3BaGabaiab=vrionaaDaaaleaacqaIWaamaeaacqWGUbGBaaaaaa@39D7@, which holds the equation

*y*·*C *= 0.     (2)

A p-invariant characterises a token conservation rule for a set of places, over which the weighted sum of tokens is constant independently from any firing, i.e., for a p-invariant *y *and any markings **m**_*i*_, **m**_*j *_∈ ℕ0n
 MathType@MTEF@5@5@+=feaafiart1ev1aaatCvAUfKttLearuWrP9MDH5MBPbIqV92AaeXatLxBI9gBaebbnrfifHhDYfgasaacH8akY=wiFfYdH8Gipec8Eeeu0xXdbba9frFj0=OqFfea0dXdd9vqai=hGuQ8kuc9pgc9s8qqaq=dirpe0xb9q8qiLsFr0=vr0=vr0dc8meaabaqaciaacaGaaeqabaqabeGadaaakeaatuuDJXwAK1uy0HMmaeHbfv3ySLgzG0uy0HgiuD3BaGabaiab=vrionaaDaaaleaacqaIWaamaeaacqWGUbGBaaaaaa@39D7@, which are reachable from **m**_0 _by the firing of transitions, it holds

*y*·**m**_*i *_= *y*·**m**_*j*_.     (3)

The nodes corresponding to the non-zero entries of an invariant *x *are called the support of *x*, written as *supp*(*x*). Considering Equations (1) and (2), it is obvious that a sum of t-invariants (p-invariants) gives again a t-invariant (p-invariant). An invariant *x *is called *minimal*, if its support does not contain the support of any other invariant *z*, i.e.,

∄ invariant *z *: *supp*(*z*) ⊂ *supp*(*x*),     (4)

and the greatest common divisor of all non-zero entries of *x *is one.

A net is covered by t-invariants (p-invariants), if every transition (place) participates in a t-invariant (p-invariant). A t-invariant (p-invariant) defines a connected subnet, consisting of its support, the support's pre- and postplaces (pre- and posttransitions), and all arcs in between.

## Methods

### Modelling

**Figure 1:**
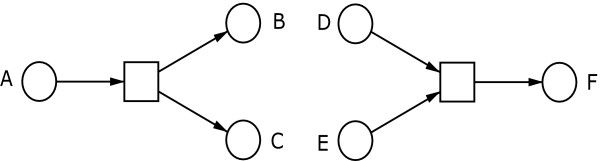
**Two conjunction subnets**. Two subnets representing conjunction-coupled implications, i.e., *A *⇒ (*B *∧ *C*) and (*D *∧ *E*) ⇒ *F*, respectively. If there is a token on place *A*, the precondition *A *is fulfilled, and the corresponding posttransition may fire. This event will fulfil the postconditions *B *and *C *getting each a token. If there is a token on the places *D *and *E*, the preconditions *D *and *E *are fulfilled, and the corresponding posttransition may fire. This event will fulfil the postcondition *F *getting a token.

First, we want to define general network components in the language of Petri nets. Considering substructures, which model logical terms as a Petri net, an ordinary implication is a trivial case. Figure 1 pictures two subnets representing conjunction-coupled implications, i.e., *A *⇒ (*B *∧ *C*) and (*D *∧ *E*) ⇒ *F*, respectively. If there is a token on place *A*, the precondition *A *is fulfilled, and the corresponding posttransition may fire. This event will fulfil the postconditions *B *and *C *getting each a token. This example illustrates that the number of tokens in a net is not generally conserved. The fulfilment of condition *F *in Figure 1 depends on both preconditions *D *and *E*.

**Figure 2:**
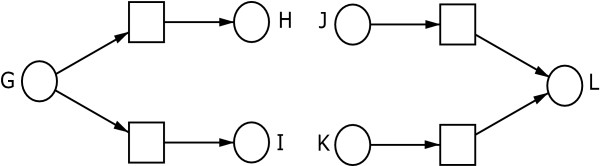
**Two disjunction subnets**. Two subnets representing disjunction-coupled implications, i.e., *G *⇒ (*H *∨ *I*) and (*J *∨ *K*) ⇒ *L*, respectively. While the subnet on the left hand side stands for an exclusive disjunction, the right-hand subnet represents an inclusive disjunction. The two posttransitions of place *G *are in conflict, i.e., if there is one token on place *G*, both its posttransitions *may *fire, but only one of them *can *actually fire. This subnet represents an exclusive disjunction. In contrast to that, the pretransitions of place *L *are concurrent and can fire independently from each other, if one token is in each of the places *J *and *K*. With this subnet an inclusive disjunction is represented.

Figure 2 depicts two subnets representing disjunction-coupled implications, i.e., *G *⇒ (*H *∨ *I*) and (*J *∨ *K*) ⇒ *L*, respectively. The two posttransitions of place *G *are in conflict, i.e., if there is one token on place *G*, both its posttransitions *may *fire, but only one of them *can *actually fire. This subnet represents an exclusive disjunction. Therefore, a nondeterministic behaviour results. In contrast to that, the pretransitions of place *L *are concurrent and can fire independently from each other, if any. With this subnet an inclusive disjunction is represented.

In all implications, presented in Figure 1 and Figure 2, the tokens in the preplaces are removed by the firing of their posttransitions. Thus, the precondition is no longer fulfilled when the connected event took place. The situation, where the state of a precondition fulfilment is preserved, is discussed below in a general form, see case 1.

**Figure 3:**
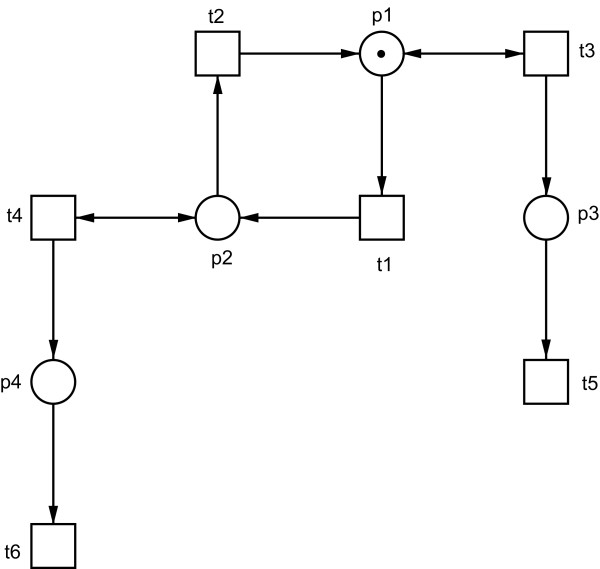
**A Petri net example**. A Petri net, whose places *p*1 and *p*2 form a p-invariant, representing for example a logical negation. *p*1 can be a condition *A *and *p*2 a condition ¬*A*, or vice versa, indicating two states of the system, for example an activated or deactivated protein. The place *p*1 carries one token to indicate in which state the system is. Transitions *t*3 and *t*4 are connected to this p-invariant by read arcs in order to hold the token on those places, which form the p-invariant.

A crucial point in signal transduction pathways are modifications of certain proteins, resulting in a functionally active or inactive form of these proteins. Many of them are activated by phosphorylation and deactivated by dephosphorylation, and act in different ways depending on their current state. A qualitative model allows by its structure to distinguish between different states of one protein. Modelling this situation, we use a subnet, which includes the different modifications (states) of one protein as places forming a p-invariant. A p-invariant *y*, which holds |*supp *(*y*)| = 2 may stand for a subnet, representing a negation by two places (*A *and ¬*A*), see Figure 3. The essential states are represented by the places *p*1 (*A*) and *p*2 (¬*A*) forming the p-invariant, i.e., the token depicted on place *p*1 circulates only between *p*1 and *p*2 by firing of *t*1 and *t*2, respectively. To preserve this invariant structure, the adjacent transitions *t*3 and *t*4, respectively, are connected via read arcs.

**Figure 4:**
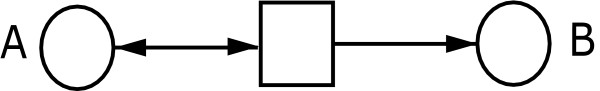
**Case 1 using read arcs**. Case 1 of using read arcs is represented. Precondition (substance) *A *does not lose its fulfilment (activity) by triggering the fulfilment (activation) of a condition (substance) *B*.

**Figure 5:**
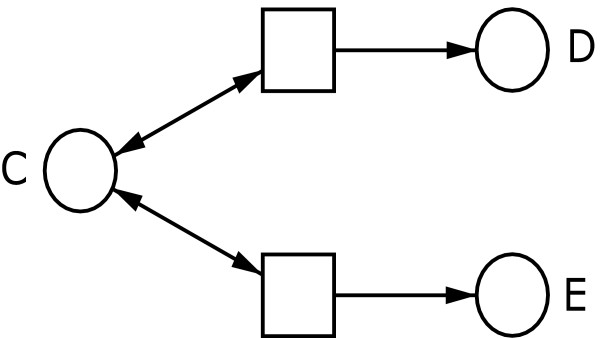
**Case 2 using read arcs**. Case 2 of using read arcs is represented. Precondition (substance) *C *triggers several events represented by transitions, which are concurrent, i.e., independent from each other.

Altogether, there are three cases in signal transduction models, which can be distinguished by their different use of read arcs:

• Case 1: A substance *A *does not lose its activity by interacting with a second substance *B*, see Figure 4.

• Case 2: A substance *C *triggers several events represented by transitions, which are independent of each other, see Figure 5.

• Case 3: The above mentioned context of a p-invariant, see Figure 3.

The subnet presented in Figure 5 differs from the subnet on the left hand side of Figure 2 by the use of read arcs. In Figure 2 the two posttransitions of place *G *are in conflict. Thus this subnet represents an exclusive disjunction *G *⇒ (*H *∨ *I*). By applying read arcs in such a substructure, the posttransitions of the corresponding place are concurrent, i.e., independent from each other. Thus, the subnet in Figure 5 represents an inclusive disjunction *C *⇒ (*D *∨ *E*).

Before turning to the next step, the model validation, let us summarise the main steps of the systematic model development:

1. compilation of biological knowledge about the signalling pathway of interest, e.g., from the literature or by database search,

2. translation of the interactions of relevant biological species into basic logical terms,

3. transferring these logical terms into net components,

4. assembling these net components to one Petri net,

5. if necessary, include input and output transitions ensuring that there are no places without pre- or posttransitions in the net.

The last but one point ensures that the developed Petri net is connected. The input and output transitions introduced in the last point make the net to a transition-bordered one. They represent the interface of the biological system to its surroundings.

### Model validation

The input transitions of a transition-bordered Petri net cause unboundedness, i.e., there is no upper bound for the number of tokens in such a net. Therefore, the state space of the net, i.e., all reachable markings, is infinite, and the dynamic net properties cannot be decided by the computation of the reachability graph, which contains all reachable markings as nodes. But, we do get information about the model dynamics via the analysis of the net structure, represented by the incidence matrix. Thus, our approach to validate the model is mainly based on the net invariants. The definition of invariants, see Equations (1) and (2), was introduced in [36]. The application of a system's minimal t-invariants to analyse metabolic networks in the steady state was proposed in [9] by the definition of elementary modes. In the context of metabolic networks p-invariants are used to model substrate conservations, see, e.g., [23,37]. However, in order to apply these techniques, which have been proven to be useful in the context of metabolic networks, to signal transduction networks, we have to adjust them first to our special needs.

P-invariants in signal transduction models represent also some kind of conservation relationship, however in the sense of the several modifications of a given species as introduced in Figure 3. A species cannot be consumed or produced, it can change its current state only, and it has to be always in exactly one state. The first step of model validation aims at checking the minimal p-invariants for their biological plausibility. Since the weighted sum of tokens over a p-invariant is constant (see Equation 3), there have to be always some tokens residing in at least one place of each minimal p-invariant ensuring that this part of the net may contribute to the system behaviour. Moreover, because a given species can always be in one state only, each p-invariant gets just one token, here. These tokens are typically placed in the p-invariants in such a way that the initial marking of the net represents an inactive state of the biological system.

The next step is to consider all possible signal flows through the network represented by realisable t-invariants, and to check their biological meaning. All possible t-invariants can be computed as non-negative linear combination of the minimal ones, see Equation (1). In former case studies, which do not contain the special p-invariant construct, see Figure 3, it was sufficient to discuss the minimal t-invariants, which are always realisable by construction [14]. Including p-invariants, we have to process the minimal t-invariants to get realisable ones, defining minimal self-contained subnets, which are active under a given input situation. For this purpose, we introduce the concept of feasible t-invariants.

### Feasible t-invariants

The calculation of t-invariants does not take into account the initial marking. Moreover, read arcs are not reflected in the incidence matrix. Therefore, the question arises, whether a minimal t-invariant is really realisable. We assume that read arcs are the only source for non-realisability, i.e., there is a sufficient token number in each of the minimal p-invariants in the initial marking.

The use of read arcs corresponding to the first case described above is discussed in [15]. For an analysis, these read arcs are replaced by unidirectional ones in the direction of the main token (signal) flow. The same rationale holds true for the replacement of the read arcs corresponding to the second case. But those ones, which are connected with (see the third case), have to remain in the net in order to keep the token-preserving structure of the p-invariant. As mentioned above, read arcs are not reflected in the incidence matrix. Therefore, depending on a given marking, some of the minimal t-invariants may not be realisable considering them in a net *with *read arcs. For example, the p-invariant-adjacent transitions *t*3 and *t*4 in Figure 3 are treated as apparent input transitions, because their connection with the places *p*1 and *p*2, respectively, is not reflected in the incidence matrix. If this kind of transitions is part of a t-invariant, whereby their preplaces (connected via read arcs) carry no tokens, they are actually not able to fire. Therefore, the minimal t-invariants have to be processed to get realisable t-invariants for a suitable marking.

In accordance with our objective of model validation, we are looking for minimal self-contained t-invariants, which are realisable in the initial marking, i.e., minimal multisets of transitions, which can fire in an appropriate order without the firing of the other transitions, not belonging to that t-invariant. Those t-invariants are called feasible t-invariants. To make a t-invariant feasible, all of its involved transitions have to be able to fire, i.e., for each of them it is true either that it is an input transition or that its preplaces enable it to fire. The latter case means that either the considered preplaces carry already sufficient tokens in the initial marking or these preplaces get sufficient tokens via the firing of other transitions involved in this t-invariant. We distinguish between realisable and feasible t-invariants, because the notion of realisability of a t-invariant is a general term of Petri net theory, referring to the reachability of a marking, where the t-invariant's transitions can fire. Feasible t-invariants are special realisable t-invariants, which are minimal ones concerning their realisability in the initial marking. They are introduced because of a special net structure, which causes the interruption of the t-invariant, when it leads for example to a p-invariant, which carries a special marking.

**Figure 6:**
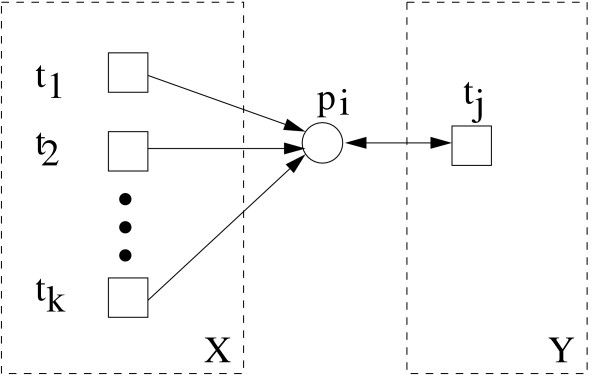
**A subnet representing the feasibility of minimal t-invariants**. The feasibility of minimal t-invariants is represented to illustrate Equation (5). Place *p*_*i *_belongs to a p-invariant. It is adjacent to the pretransitions *t*_1_, *t*_2_,...*t*_*k *_and connected to the transition *t*_*j *_by a read arc. *X *and *Y *stand for the sets of t-invariants including the pre- and the posttransitions, respectively, which are contained in the corresponding dashed rectangles. Since *p*_*i *_does not carry tokens, t-invariants are not feasible if they contain transition *t*_*j *_without containing at least one of the transitions *t*_1_, *t*_2_,...*t*_*k *_. Thus, the read arc has to be bridged as described in Equation (5) to get feasible t-invariants.

Table 1 lists all feasible t-invariants of the net in Figure 3, consisting of all minimal t-invariants and one additional processed one. The processing of minimal t-invariants looks for minimal (non-negative) linear combinations, resulting into feasible t-invariants *z*. Let us formulate this processing of the minimal t-invariants in a general form. For each read arc between a transition *t*_*j *_and an empty place *p*_*i *_of a p-invariant the following procedure has to be applied. Let *t*_1_,...,*t*_*k *_be all the pretransitions of *p*_*i*_, which are not connected via a read arc with *p*_*i*_, see Figure 6. If there are no tokens residing in *p*_*i *_in the initial marking, a t-invariant is not realisable if it includes *t*_*j *_and excludes *t*_*l *_∀ *l *∈ {1,..., *k*}. The following combinations *z *of feasible t-invariants *x *and one non-feasible t-invariant *y *reestablish the given read arc between transition *t*_*j *_and place *p*_*i*_

**Table 1:** The t-invariants of the example in Figure 3.

No.	Involved transitions	Minimal?	Feasible?	Composed of
1.	1, 2	√	√	-
2.	3, 5	√	√	-
3.	4, 6	√	-	-
4.	1, 2, 4, 6	-	√	3+1

The t-invariants of the net in Figure 3 after processing them. The middle columns indicate, whether the t-invariants are minimal, i.e., non-processed and whether they are feasible. The right column indicates how the processed invariant was built.

*z *= *x *+ *y *∀*x *∃ *l *∈ {1,...,*k*} : *x*_*l *_≠ 0

with *x*_*j *_= 0

and *y*_*j *_≠ 0 ∧ ∀ *l *∈ {l,...,*k*} : *y*_*l *_= 0.     (5)

Note that for each feasible minimal t-invariant *x *providing a token on the place *p*_*i *_one linear combination *z *is constructed. Appropriate combinations have to be performed iteratively, if there occur several read arcs in the net, which have to be bridged. The combined t-invariants do not hold the criterion to be minimal, see Equation (4). But, since only inevitable combinations of minimal t-invariants are constructed, see the restrictions in Equation (5), the resulting combined t-invariants are minimal with respect to their realisability in the initial marking. Moreover, they only have non-negative entries, because they are build as a sum of two t-invariants, which are defined as vectors in ℕ0m
 MathType@MTEF@5@5@+=feaafiart1ev1aaatCvAUfKttLearuWrP9MDH5MBPbIqV92AaeXatLxBI9gBaebbnrfifHhDYfgasaacH8akY=wiFfYdH8Gipec8Eeeu0xXdbba9frFj0=OqFfea0dXdd9vqai=hGuQ8kuc9pgc9s8qqaq=dirpe0xb9q8qiLsFr0=vr0=vr0dc8meaabaqaciaacaGaaeqabaqabeGadaaakeaatuuDJXwAK1uy0HMmaeHbfv3ySLgzG0uy0HgiuD3BaGabaiab=vrionaaDaaaleaacqaIWaamaeaacqWGTbqBaaaaaa@39D5@, see Equation (1).

Feasible t-invariants define, similarly to the t-invariants, connected subnets, characterised by its support. Generally, these subnets overlap. They represent minimal self-contained subnets being active under a given input situation. So, each of these subnets stands for a possible signal flow in the net. The examination of the feasible (minimal or combined) t-invariants for their biological plausibility is a central step of model validation. It has to be checked, whether each feasible t-invariant has a biological meaning and whether every modelled part of the considered signalling pathway is reflected in a corresponding feasible t-invariant [14].

Finally, it has to be checked, whether the net is covered by t-invariants. This property ensures that every transition participates in a t-invariant, i.e., every biological atomic action in the model may take place as part of the basic behaviour of the net.

While minimal t-invariants are computed based on the net structure only, feasible t-invariants are constructed with respect to the marking of the net. Summarising the discussion above, the following situations are possible: the minimal t-invariants can reach (1) from input to output transitions, (2) from input transitions to minimal p-invariants, (3) from minimal p-invariants to minimal p-invariants, or (4) from minimal to output transitions. The transitions of a t-invariant influence a minimal p-invariant in such a way that altogether its initial state is reproduced. Not every possible combination is considered to bridge a read arc and to connect minimal t-invariants. Such a procedure would lead to t-invariants reaching from input transitions to output transitions, only. As described above, those minimal t-invariants are combined, which *meet *at a minimal p-invariant with a corresponding place not carrying a token.

### Maximal common transition sets (MCT-sets)

In order to support the check of feasible t-invariants for their biological meaning, the transitions are grouped into so-called *maximal common transition sets *(*MCT-sets*) by their occurrence in the minimal t-invariants: ∀*i*, *j *∈ {1,...,*m*} the transitions *t*_*i *_and *t*_*j *_are grouped into the same MCT-set, if and only if they participate in exactly the same minimal t-invariants, i.e., all t-invariants *x *hold

*χ*_{0}_(*x*_*i*_) = *χ*_{0}_(*x*_*j*_),     (6)

whereas *χ*_{0} _denotes the characteristic function, binary indicating if an argument is equal to zero. This support-oriented grouping leads to maximal sets of transitions, where each set of transitions *ϑ *holds

∀*x *∈ *X *: *ϑ *⊆ *supp*(*x*) ∨ *ϑ *∩ *supp*(*x*) = ∅,     (7)

whereas *X *denotes the set of all minimal t-invariants *x*.

The grouping according to Equation (6) represents an equivalence relation in *T*, the set of transitions, which leads to a partition of *T*. The equivalence classes *ϑ *are the MCT-sets. Transitions, not contained in any t-invariant, form their own MCT-set. MCT-sets define subnets, but not necessarily connected ones. An example of a disconnected MCT-set will be given below in the presented model.

The resulting MCT-sets are disjunctive and represent a possible decomposition of large biochemical networks into rather small subnets, which can be read as functional units. Because of the way, in which they are generated, each of the MCT-sets may represent a signalling unit or a building block with its own biological meaning. In the following, only those MCT-sets are considered, which contain more than one transition.

## Results and discussion

### Derivation of the Petri net model

**Figure 7:**
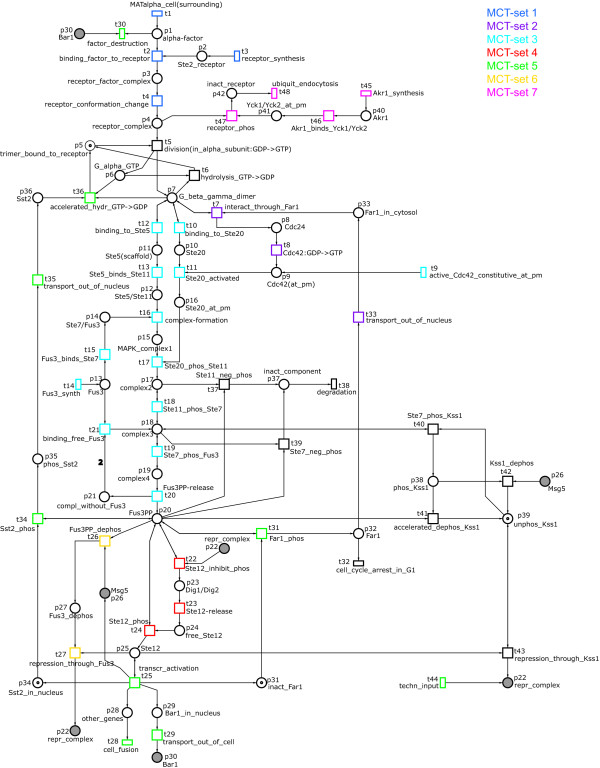
**The Petri net model of the mating pheromone response pathway of S. cerevisiae**. The Petri net, modelling the signal transduction pathway of the mating pheromone response in *S. cerevisiae*. The meaning of the places is listed in Table 3 and of the transitions in Table 4. The logical nodes are coloured in grey. A logical node is identified by its name and exists in multiple copies in the net, which are logically identical. This construct is mainly used to avoid immoderate arc crossings. The transitions, which are contained in the seven MCT-sets of Table 5, are coloured differently.

In accordance with the elucidation above we developed a Petri net, modelling the signal transduction pathway of the mating pheromone response in *S. cerevisiae*. The Petri net in Figure 7 presents – to the best of our knowledge – for the first time a qualitative model of the pheromone pathway, and it extends the ODE model given in [6]. The net consists of 42 places and 48 transitions, which are listed by their name and biological meaning in Tables 3 and 4, respectively.

**Table 3:** The places of the model.

ID	Place name	Biological species
1	alpha-factor	pheromone released by an MAT*α *cell in the surroundings
2	Ste2 _receptor	mating pheromone receptor of the modelled MAT**a **cell
3	receptor_ factor_complex	complex consisting of the *α*-factor and the Ste2 receptor
4	receptor_complex	the above named complex is activated by a conformation change
5	trimer_bound_to_receptor	heterotrimeric G protein, which is coupled to the Ste2 receptor
6	G_alpha_GTP	dissociated G*α *subunit (exchange of GDP to GTP in this monomer)
7	G_beta_gamma_dimer	G-protein G*βγ *subunits in a dimeric form
8	Cdc24	Cdc24, i.e., guanine nucleotide exchange factor of Cdc42
9	Cdc42(at_pm)	Cdc42 located at the plasma membrane
10	Ste20	protein kinase Ste20
11	Ste5 (scaffold)	Ste5, acting as a scaffold protein
12	Ste5/Ste11	protein complex consisting of Ste5 and Ste11
13	Fus3	MAP kinase Fus3
14	Ste7/Fus3	protein complex consisting of Ste7 and Fus3
15	MAPK_complex1	MAPK complex consisting of Ste5, Ste11, Ste7 and Fus3
16	Ste20_at_pm	Ste20 located at the plasma membrane, i.e., near the MAPK complex
17	complex2	as complex1, but Ste11 is activated additionally
18	complex3	as complex2, but Ste7 is activated additionally
19	complex4	as complex3, but Fus3 is activated additionally
20	Fus3PP	dissociated Fus3 in the activated form
21	compl_without_Fus3	as complex4, but without Fus3
22	repr_complex	complex containing Ste12 repressed by Fus3 or Kss1 and Dig1/Dig2
23	Dig1/Dig2	Ste12 inhibitors, i.e., cofactors for the repression
24	free_Ste12	Ste12 released out of the repression complex
25	Ste12	activated transcription factor Ste12
26	Msg5	phosphatase Msg5 being able to deactivate Fus3 or Kss1
27	Fus3_dephos	deactivated Fus3
28	other_genes	pheromone regulated genes encoding mating related cell responses
29	Bar1_in_nucleus	synthesised protease Bar1 located in the nucleus
30	Bar1	Bar1 secreted in the cell environment
31	inact_Far1	synthesised Far1 located in the nucleus in an inactive form
32	Far1	Far1 activated by phosphorylation
33	Far1_in_cytosol	active Far1 located in the cytosol
34	Sst2_in_nucleus	synthesised Sst2 located in the nucleus in an inactive form
35	phos_Sst2	Sst2 activated by phosphorylation
36	Sst2	active Sst2 located in the cytosol
37	inact_component	complex labelled for degradation by phosphorylation
38	phos_Kss1	MAP kinase Kss1 activated by phosphorylation
39	unphos_Kss1	inactive Kss1
40	Akr1	protein Akr1 located at plasma membrane
41	Yck1/Yck2_at_pm	kinases Yck1/Yck2 being able to label the Ste2 for degradation
42	inact_receptor	receptor labelled for ubiquitination and endocytosis

The places of the model, see Figure 7, each listed with its name, its ID and the biological species represented by this node.

**Table 4:** The transitions of the model.

ID	Transition name	Biological event
1	MATalpha_cell(surroundings)	a near MAT*α *cell secretes its mating pheromone
2	binding_factor _to_receptor	the *α*-factor binds to the Ste2 receptor
3	receptor_synthesis	synthesis of the cell surface receptor Sst2
4	receptor _conformation_change	conformation change of the receptor
5	division(in_alpha_subunit:GDP->GTP)	dissociation of the G*α *subunit of the G-protein
6	hydrolysis_GTP->GDP	hydrolysis reassociates G*α *with G*βγ*
7	interact_through_Far1	G*βγ *interacts Far1 transmitted with Cdc24
8	Cdc42:GDP->GTP	Cdc24 supported activation of Cdc42
9	active_Cdc42_constitutive_at_pm	constitutive active Cdc42 attending the processes
10	binding_to_Ste20	G*βγ *binds Ste20
11	Ste20_activated	Cdc42 at plasma membrane activates Ste20
12	binding_to_Ste5	G*βγ *binds Ste5
13	Ste5_binds_Ste11	Ste5 binds Ste11
14	Fus3_synth	synthesis of kinase Fus3
15	Fus3_binds_Ste7	Ste7 binds Fus3
16	complex-formation	Ste5/Ste11 binds Ste7/Fus3
17	Ste20_phos_Ste11	phosphorylation of Ste11 by Ste20
18	Ste11_phos_Ste7	phosphorylation of Ste7 by Ste11
19	Ste7_phos_Fus3	phosphorylation of Fus3 by Ste7
20	Fus3PP-release	release of activated Fus3 out of the MAPK complex
21	binding_free_Fus3	remaining MAPK complex binds Fus3
22	Ste12_inhibit _phos	phosphorylation of Ste12 inhibitors Dig1/Dig2 by Fus3PP
23	Ste12-release	release of Ste12 out of the repression complex
24	Ste12_phos	phosphorylation of Ste12 by Fus3PP
25	transcr_activation	transcription activation of pheromone regulated genes
26	Fus3PP_dephos	dephosphorylation of Fus3PP by Msg5
27	repression_through_Fus3	Ste12 repression through inactive Fus3 and Dig1/Dig2
28	cell_fusion	processes leading to the fusion of the two haploid cells
29	transport_out_of_cell	Bar1 transport into the cell environment
30	factor _destruction	Bar1 transmitted destruction of the *α*-factor
31	Far1_phos	phosphorylation of Far1 by Fus3PP
32	cell_cycle_arrest in_G1	Far1 caused arrest in the cell cycle phase G1
33	transport_out_of_nucleus	Far1 transport out of the nucleus
34	Sst2_phos	phosphorylation of Sst2 by Fus3PP
35	transport_out_of_nucleus	Sst2 transport out of the nucleus
36	accelerated_hydr_GTP->GDP	accelerated hydrolysis reassociates the G-protein
37	Ste11_neg_phos	Fus3PP labels the MAPK complex at Ste11 for degradation
38	degradation	degradation of the MAPK complex
39	Ste7_neg_phos	Fus3PP labels the MAPK complex at Ste7 for degradation
40	Ste7_phos_Kss1	phosphorylation of Kss1 by Ste7
41	accelerated-dephos_Kss1	deactivation of phosphorylated Kss1 by Fus3PP
42	Kss1_dephos	dephosphorylation of phosphorylated Kss1 by Msg5
43	repression_through_Kss1	Ste12 repression through inactive Kss1 and Dig1/Dig2
44	techn_input	technical: the repressed Ste12 complex assumed to be present
45	Akr1_synthesis	synthesis of Akr1
46	Akr1_binds_Yck1/Yck2	Akr1 binds Yck1/Yck2
47	receptor_phos	labelling of Ste2 for degradation
48	ubiquit_endocytosis	ubiquitination and endocytosis of the receptor

The transitions of the model, see Figure 7, each listed with its name, its ID and the biological event represented by this node.

Proteins, protein complexes, and other chemical compounds are represented as places, and complex formation/cleavage, protein de-/phosphorylation, and other reactions by transitions. Tables 3 and 4 show in their right column, which chemical compounds and which biological events are considered in the model. Generally formulated, the model contains a complete pheromone response pathway, i.e., activation and composition of the pheromone receptor complex, composition of the MAP kinase cascade complex and MAP kinase cascade, transcription factor activation, and transcription of genes, which response to the mating pheromone. Moreover, there are several positive and negative feedback regulations included in the model, i.e., regulation of the ligand *α*-factor (via Bar1), of the receptor Ste2 (endocytosis induced by Yck1 and Yck2), of G-protein subunits (via Sst2), of the MAPK cascade (via labelling for degradation), and of the regulation of transcription (via repression of the transcription factor Ste12).

We take into account the above mentioned substructures, e.g., the place *complex2 *(*p*17) does not lose its activity by firing of transition *Ste11_phos_Ste7 *(*t*18), i.e., in the MAPK-complex the protein Ste11 remains in its active form after activating the protein Ste7, see case 1. The place *Fus3PP *(*p*20) triggers several transitions *Ste12_inhibit_phos t*22), *Ste12_phos *(*t*24), *Far1-phos *(*t*31), and *Sst2_phos *(*t*34), which are independent of each other, see case 2. The place pair *Kss1_phos *(*p*38) and *unphos_Kss1 *(*p*39) forms a p-invariant as discussed in case 3.

With one exception, there exist only arcs weighted by 1 because no substance flow with stoichiometric relations takes place, but signal flow is described by the model. This special arc is weighted by 2 to reduce the token number in the structural cycle, which is formed by the places *complex3 *(*p*18), *complex4 *(*p*19), *compl_without_Fus3 *(*p*21), and the transitions *Ste7_phos_Fus3 *(*t*19), *Fus3PP-release *(*t*20), and *binding_free_Fus3 *(*t*21). By using this arc weight, it is ensured that the activation of the transition *Ste7_phos_Fus3 *(*t*19) depends on firing of transition *Ste11_phos_Ste7 *(*t*18), see Figure 7. In the biological context, this means that the crucial phosphorylation of the last kinases in the MAPK cascade depends on the activation of the last but one.

In the initial marking there is one token residing in the place *inact_Far1 *(*p*31) and *Sst2_in_nucleus *(*p*34), respectively, which symbolise that these proteins are already present in the cell in low concentration independently of a signal.

### Application of Petri net concepts

#### P-invariant analysis

There are three minimal p-invariants in the net. Table 2 contains these p-invariants as well as the corresponding biological meaning of them. P-invariants 1 and 2, respectively, stand for the modification of the dimeric and monomeric, respectively, subunits of the receptor coupled G-protein, which occur either in the trimeric form represented in the place *trimer_bound_to_receptor *(*p*5) or which is dissociated in its subunits *G_beta_gamma_dimer *(*p*7) and *G_alpha_GTP *(*p*6). Therefore, both of these p-invariants include the place *p*5 in addition to one of the places *p*6 and *p*7, respectively. The places *Kss1_phos *(*p*38) and *unphos_Kss1 *(*p*39) indicate the modification of the involved kinase Kss1 and form the third minimal p-invariant according to case 3. Thus, all these minimal p-invariants can be interpreted in a biologically reasonable meaning.

**Table 2:** The minimal p-invariants and their biological meaning.

No.	Places	Biological meaning
1.	5, 7	G*βγ *subunits: dimer and bound in G protein trimer
2.	5, 6	G*α *subunit: monomer and bound in G protein trimer
3.	38, 39	Kss1: active and inactive form

The minimal p-invariants and their biological meaning.

In the initial marking of the net there are some tokens already residing in special places. According to the elucidation in section *Model validation*, the places *trimer_bound_to_receptor *(*p*5) and *unphos_Kss1 *(*p*39) carry tokens ensuring that the minimal p-invariants contribute to the system behaviour.

#### T-invariant analysis

For our model we got ten minimal t-invariants covering all transitions. As mentioned above, the transitions are grouped into MCT-sets, if and only if they participate in exactly the same minimal t-invariants, see Equation (6). If a huge number of t-invariants arises for a network, the concept of MCT-sets provides additional structure information. Due to their definition, MCT-sets represent sets of events, which occur only together. These sets, together with their pre- and postplaces and all arcs in between, should symbolise signalling functional units of the model with an own biological meaning, see Table 5. For example, MCT-set 1 consists of the transitions *MATalpha_cell*(*surrounding*) (*t*1), *receptor_synthesis *(*t*3), *binding_factor_to_receptor *(*t*2), and *receptor_conformation_change *(*t*4). Therewith, this MCT-set represents the synthesis of Ste2, its binding to the present *α*-factor and its resulting conformation change. In Figure 7 the MCT-sets, which contain more than one transition, are shown in different colours. As mentioned above, the MCT-sets are not necessarily connected subnets. MCT-set 5 gives an example for a disconnected one because it contains *techn_input *(*t*44,), compare Table 5 and Figure 7.

**Table 5:** The maximal common transition sets.

MCT-set	Transitions	Biological meaning
1	1, 2, 3, 4	Receptor activation
2	7, 8, 33	Interaction G*βγ*-Cdc24 via Far1
3	9, 10, 11, 12, 13, 14, 15, 16, 17, 18, 19, 20, 21	MAP kinase cascade
4	22, 23, 24	Ste12 activation
5	25, 28, 29, 30, 31, 34, 35, 36, 44	Transcription of the pheromone-responding genes
6	26, 27	Ste12 repression through inactive Fus3
7	45, 46, 47, 48	Receptor endocytosis

Transitions grouped into maximal common transition sets over their occurrence in the same feasible t-invariants. The right column contains the biological meaning of the resulting set. Only MCT-sets with more than one transition are considered here

The necessarily in the net remaining read arcs endanger the feasibility, i.e., the realisability in the initial marking, of the minimal t-invariants. Following our modelling approach, minimal t-invariants are not feasible, if they involve a transition, which is connected via a read arc with an empty preplace, which does not get tokens by firing of other transitions involved in this t-invariant. To ensure the feasibility of all t-invariants, these non-feasible ones are joint with some others, which provide tokens on the critical preplaces. The read arcs adjacent to the place *G_beta_gamma_dimer *(*p*7) remain in the net because of the minimal p-invariant 1. Therefore, the minimal t-invariants 3, 4, 5, and 6 do not fulfil the feasibility criterion, because they contain the MCT-set 3, but do not contain any transitions, which provide tokens at place *p*7, which is connected with the MCT-set 3 via read arcs. These non-feasible t-invariants have to be processed in the way explained above, see Equation (5). Only the minimal t-invariant 1 involves transition *division *(*in_alpha_subunit:GDP→GTP*) (*t*5), which provides tokens on place *p*7 and does not involve the MCT-set 3. Therefore, each of the four non-feasible t-invariants is combined with t-invariant 1. These combinations provide four feasible, non-minimal t-invariants. Table 6 lists the feasible t-invariants and indicates if they are still minimal, i.e., non-processed.

**Table 6:** The t-invariants.

No.	Involved transitions		Minimal?	Feasible?	Composed of
	MCT-sets	single transitions			
1.	1	5, 6	√	√	-
2.	1, 7	-	√	√	-
3.	3	37, 38	√	-	-
4.	3	38, 39	√	-	-
5.	3, 4	43	√	-	-
6.	3	40, 41	√	-	-
7.	1, 3, 4, 5	5, 32, 40, 42	√	√	-
8.	1, 2, 3, 4, 5	5, 40, 42	√	√	-
9.	1, 3, 4, 5, 6	5, 32	√	√	-
10.	1, 2, 3, 4, 5, 6	5	√	√	-
11.	1, 3	5, 6, 37, 38	-	√	3 + 1
12.	1, 3	5, 6, 38, 39	-	√	4 + 1
13.	1, 3, 4	5, 6, 43	-	√	5 + 1
14.	1, 3	5, 6, 40, 41	-	√	6 + 1

The t-invariants of the net after their processing. The middle columns show, whether the invariants are minimal and/or feasible. The right column indicates how the processed invariants were built.

We want to discuss the biological plausibility of the feasible t-invariants. Table 7 contains the biological meaning of the t-invariants in Table 6. They all include the receptor synthesis and the binding of the pheromone factor of a MAT*α *cell in the surroundings, causing a receptor conformation change (MCT-set 1). In t-invariant 1 the activated receptor causes the dissociation of the G-protein (transition *t*5), whose subunits reassociate to a trimeric form (transition *t*6). In the t-invariant 2 the proteins Yck1 and Yck2 are bound through Akr1 to the plasma membrane and phosphorylate the activated receptor. This leads to a receptor endocytosis (MCT-set 7) via an ubiquitination. The t-invariants 7 to 14 include a signal transduction via the G-protein (transition *t*5) and the MAP kinase cascade (MCT-set 3). A release and phosphorylation of the transcription factor Ste12 (MCT-set 4) is involved in the t-invariants 7 to 10. This leads in these t-invariants to a transcription of the pheromone response genes preparing the cell for mating, including a feedback via Bar1 and Sst2 (MCT-set 5). Far1 is transported out of the nucleus (MCT-set 2) in t-invariants 8 and 10, while it initiates the cell cycle arrest (transition *t*32) in the t-invariants 7 and 9. By containing MCT-set 6, the t-invariants 9 and 10 include a Ste12 repression via activated Fus3. In t-invariants 7 and 8, kinase Kss1 is activated and deactivated (transitions *t*40 and *t*42, respectively). This p-invariant 3 contributes also to the processes described in t-invariant 14, where Kss1 is phosphorylated and dephosphorylated (through transitions *t*40 and *t*41, respectively). Generally, the t-invariants 11 to 14 represent signalling pathways, which do not lead to a mating of the cell. All these t-invariants consist only of the receptor activation (MCT-set 1) and the MAP kinase cascade (MCT-set 3). Only t-invariant 13 involves an additional activation of Ste12 (MCT-set 4), which is repressed via Kss1 (transition *t*43). The t-invariants 11 and 12 include a feedback inhibition via a protein degradation (transition *t*38), which is initiated through a phosphorylation of these proteins (transitions *t*37 and *t*39, respectively).

**Table 7:** The biological meaning of the feasible t-invariants.

No.	Biological meaning
1.	Dissociation and reassociation of the G protein subunits
2.	Endocytosis of the activated receptor
7.	Changed gene transcription, cell cycle arrest, de-/phosphorylation Kss1
8.	Changed gene transcription, Far1 transport out of the nucleus, de-/phosphorylation Kss1
9.	Changed gene transcription, cell cycle arrest, repression of Ste12 through inactive Fus3
10.	Changed gene transcription, Far1 transport out of the nucleus, repression of Ste12 through inactive Fus3
11.	Signalling via the cascade, feedback degradation of Ste11
12.	Signalling via the cascade feedback degradation of Ste7
13.	Signalling via the cascade, repression of Ste12 through inactive Kss1
14.	Signalling via the cascade, phosphorylation and dephosphorylation of Kss1

The feasible t-invariants of Table 6 with their biological meaning. All of them involve the receptor activation, which is not explicitly mentioned, here.

Recapitulating, t-invariants 7 to 10 lead to a mating of the cell, while the other t-invariants enable the cell to regulate, and modulate a pheromone induced signal by attenuated response or response desensitisation.

Altogether it can be summarised that the known signalling processes are represented in the feasible t-invariants.

### Theoretical knockout experiments

The Petri net model can serve as basis of theoretical knockout experiments. They can be constructed by deleting the corresponding place and adjacent transitions, modelling the absence of this compound in a null mutant. Animating the resulting net shows already where some token accumulations arise under the new situation. These affected compounds can characterise a null mutant. Theoretical knockout experiments by the Petri net model could point out, which compounds indicate a mutant and nominate possible indicators for biological exploration.

Furthermore, a deletion of transitions leads to a deletion of feasible t-invariants, at least in a net, which is covered by t-invariants. The remaining feasible t-invariants show, which events still take place in this new cell type. For example, it is known [30] that Ste5 null mutant cells are unable to mate or to arrest the cell cycle. This experiment can be reconstructed in our Petri net model by deleting the place *Ste5*(*scaffold*) (*p*11) and its adjacent transitions *binding_to_Ste5 *(*t*12) and *Ste5_binds_Ste11 *(*t*13). This results in the deletion of the MCT-set 3, see Table 5. The feasible t-invariants 7 to 14 do not exist in the new mutant net because they include MCT-set 3, compare Table 6. The transitions *cell_fusion *(*t*28) (i.e., MCT-set 5), and *celI_cycle_arrest_in_G1 *(*t*32) occur only in the feasible t-invariants 7 to 10, which were deleted. Thus, the Ste5 mutant cells modelled by that mutant net are neither able to arrest the cell cycle nor to undergo a cell fusion, because the corresponding transitions do not occur in the feasible t-invariants of the net.

## Conclusion

In this paper we describe a systematic approach to model and analyse signal transduction pathways using qualitative Petri nets. We propose a step-wise model development via the translation of the biological interactions into logical terms, which then in turn are transferred into net components.

We explain the model validation step with a strong focus on the invariant analysis. In our modelling approach, we use minimal p-invariants, e.g., for the representation of the switching behaviour representing a compound in its activated and deactivated form. These p-invariants are embedded into the whole network by read arcs. Therefore, minimal t-invariants have generally to be processed to get feasible t-invariants. According to the construction principle, feasible t-invariants correspond to minimal self-contained subnets active under a given input situation. So, feasible t-invariants have to be checked for their biological meaning.

Furthermore, we define maximal common transition sets, which provide a decomposition of the net into not necessarily connected subnets. These structures can be interpreted as the smallest biologically meaningful functional units or building blocks of a given network.

The new concepts of feasible t-invariants and MCT-sets have been proven to be useful for model validation and the interpretation of the biological system behaviour by their fully automatic approach to decompose a given network into biologically meaningful modules. Please note that it is possible to build the MCT-sets based on the feasible t-invariants instead of the minimal ones. That makes sense in Petri nets, which contain more substructures of minimal p-invariants. MCT-sets provide a decomposition of the net into disjunctive subnets, and feasible t-invariants describe subnets, which can overlap.

We illustrate this approach using the mating pheromone pathway in *S. cerevisiae*. The validated model denotes the known signal flows through the net, which are obtained by the invariant analysis. We are able to assign a reasonable biological meaning to all feasible t-invariants. These reflect correctly the signal transduction in such a system, i.e., the signal response behaviour. Thus, Petri net theory is also useful to model and analyse signalling pathways considering not a substance flow, but a flow of information (signals) through the net.

The Petri net model may serve as basis for theoretical knockout experiments. By deleting appointed places and adjacent transitions, null mutants can be modelled. The analysis results of the original net can be easily modified to get some information about the derived mutant cell.

Having once validated the structure of the qualitative discrete Petri net, several applications and extensions are possible. The net may be refined and extended to a quantitative one by including known or estimated kinetic parameters (e.g., as concentrations, reaction rates, equilibrium constants), using continuous or hybrid Petri nets, respectively [38,39].

In this way, the resulting quantitative Petri net preserves the structure of the underlying net topology. Continuous Petri nets may be considered as a structured ODEs description. They can be evaluated by standard differential equation solvers. For a related case study see [40]. Hybrid Petri net comprise generally discrete as well as continuous parts. Their evaluation requires dedicated simulation approaches. For a related case study see [24]. In both cases, the quantitative simulation will allow the observation of signal flows, already revealed by the underlying discrete model. However, contrary to our qualitative approach, where signal flows are represented explicitly by the computed feasible t-invariants, signal flows in the quantitative model are given only implicitly by the simulation results.

In this paper, we have chosen deliberately a rather small running example to be able to present all steps of the analysis procedure in full detail, which should help the reader to mimic the approach using his/her own case studies. It would be worth applying our approach to larger networks as presented in [41,42].

### Software tools and supplementary material

The development of the net has been done using the graphical Petri net editor and animator Snoopy [43] described in [44]. The analyses have been performed using the Integrated Net Analyzer INA [45]. Both tools are freely available. They are running under Windows as well as under Unix/Linux. We provide the Petri net model and the INA analysis result file in the supplementary material [46].

## Authors' contributions

The paper is a product of a strong co-operation between all three authors. The key concepts and case study has been elaborated by AS during the work on her diploma thesis. MH gave substantial and valuable contributions in the application of Petri net techniques. IK defined the topic and gave substantial contributions to the conceptional design of the research project. The editorial work has been done by all three authors. All authors read and approved the final manuscript.
